# Suppression of NLRP3 Inflammasome Activation Ameliorates Chronic Kidney Disease-Induced Cardiac Fibrosis and Diastolic Dysfunction

**DOI:** 10.1038/srep39551

**Published:** 2016-12-21

**Authors:** Antoinette Bugyei-Twum, Armin Abadeh, Kerri Thai, Yanling Zhang, Melissa Mitchell, Golam Kabir, Kim A. Connelly

**Affiliations:** 1 Keenan Research Centre for Biomedical Science, St. Michael’s hospital, Toronto, Ontario, Canada; 2 Institute of Medical Science, University of Toronto, Toronto, Ontario, Canada; 3 Division of Cardiology, St. Michael’s hospital, Toronto, Ontario, Canada

## Abstract

Cardiac fibrosis is a common finding in patients with chronic kidney disease. Here, we investigate the cardio-renal effects of theracurmin, a novel formulation of the polyphenolic compound curcumin, in a rat model of chronic kidney disease. Briefly, Sprague-Dawley rats were randomized to undergo sham or subtotal nephrectomy (SNx) surgery. At 3 weeks post surgery, SNx animals were further randomized to received theracurmin via once daily oral gavage or vehicle for 5 consecutive weeks. At 8 weeks post surgery, cardiac function was assessed via echocardiography and pressure volume loop analysis, followed by LV and renal tissue collection for analysis. SNx animals developed key hallmarks of renal injury including hypertension, proteinuria, elevated blood urea nitrogen, and glomerulosclerosis. Renal injury in SNx animals was also associated with significant diastolic dysfunction, macrophage infiltration, and cardiac NLRP3 inflammasome activation. Treatment of SNx animals with theracurmin improved structural and functional manifestations of cardiac injury associated with renal failure and also attenuated cardiac NLRP3 inflammasome activation and mature IL-1β release. Taken together, our findings suggest a significant role for the NLRP3 inflammasome in renal injury-induced cardiac dysfunction and presents inflammasome attenuation as a unique strategy to prevent adverse cardiac remodeling in the setting of chronic kidney disease.

Despite considerable progress in the treatment of chronic kidney disease, cardiovascular complications remain a major cause of mortality amongst patients^1^. In fact, it is thought that 90% of patients with chronic kidney disease will die of cardiovascular complications before progressing to end-stage renal failure^2^, suggesting that current treatment options do not adequately address the underlying mechanisms driving the increase in cardiovascular mortality. Clearly, there exists a need for the development of novel therapies that provide a cardio-protective benefit for patients with chronic kidney disease.

Left ventricular hypertrophy and interstitial cardiac fibrosis are common structural changes in the heart that occur during renal failure^3^,^4^. Although studies have reported that these changes stem from a response to hormonal (renin-angiotensin-aldosterone system) and hemodynamic (high blood pressure) stimulus, the involvement of inflammatory mechanisms is often overlooked. Renal injury often initiates an intense inflammatory response that promotes dysfunction and pathological remodeling in the heart. The NOD-like receptor family protein NLRP3 is an intracellular signaling molecule that acts as a danger signal sensor and becomes activated in response to tissue damage, metabolic stress, and infection^5^. When activated, NLRP3 recruits the apoptosis associated speck-like protein containing a caspase recruitment domain (ASC), which interacts with caspase-1 to form a multiprotein complex termed the “inflammasome”. The formation of the inflammasome leads to the activation of caspase-1, which cleaves pro-inflammatory cytokines IL-1β and IL-18 to their active and secreted forms. To date, several studies in humans and animal models of kidney disease have reported elevated levels of NLRP3, IL-1β and/or IL-18; thus defining the NLRP3 as a critical component of the inflammatory process in kidney disease^6^,^7^,^8^.

The role of the NLRP3 inflammasome in kidney disease is further emphasized by a recent study which showed that loss of NLRP3 significantly reduced inflammation and tubulointerstitial fibrosis in mice after unilateral ureteral obstruction, a relevant model of chronic kidney disease^9^. Similarly, cardiac-related complications have also been shown to be mediated by NLRP3^10^. Indeed, the formation of the inflammasome in the myocardium has been shown to lead to adverse cardiac remodeling and caspase-1 mediated cell death.

Curcumin (diferuloylmethane) is an active component of the spice turmeric that has been shown to have antioxidant and anti-inflammatory properties. To date, curcumin has been reported to be both renal and cardiac protective and has also been shown to suppress acute and chronic inflammation. However, because of its poor solubility and oral bioavailability, the application of curcumin in the clinic has been quite limited^11^. To overcome this, a novel formulation of curcumin—with improved solubility and oral bioavailability—was developed, named theracurmin^12^. Here, we report that theracurmin reduces cardiac fibrosis and improves diastolic function in a rat model of chronic kidney disease. We show that theracurmin attenuates NLRP3 inflammasome activation in the heart and reduces circulating IL-1β levels, illustrating a cardio-protective effect of the compound and the potential therapeutic benefits of a theracurmin-based treatment strategy in the setting of chronic kidney disease.

## Results

### Theracurmin improves systolic blood pressure in SNx animals

**Figure 1:**
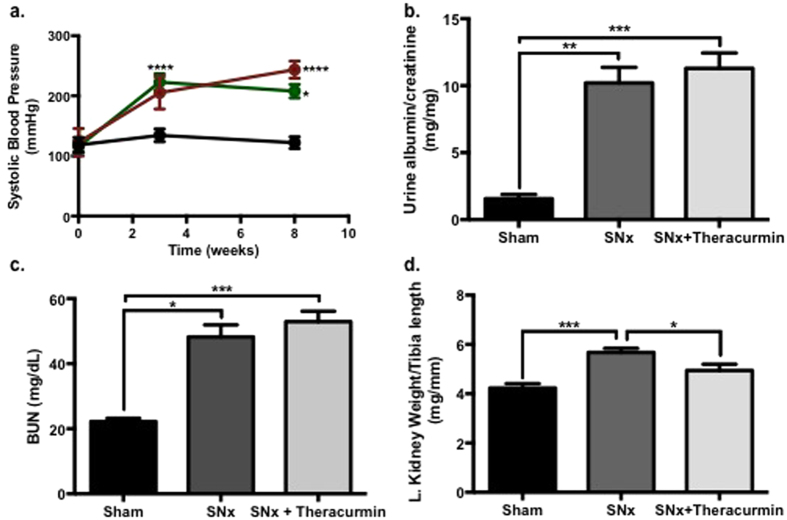
Theracurmin reduces systolic blood pressure in SNx animals. (**a**) Systolic blood pressure in sham-operated animals (black line), SNx (red line) and theracurmin-treated SNx animals (green line). (**b**) Urine albumin:creatinine ratio, (**c**) blood urea nitrogen and (**d**) left kidney weight in sham-operated controls, SNx, and theracurmin-treated animals at eight weeks post surgery. Data presented as mean ± SEM. *p < 0.05, **p < 0.01, ***p < 0.001, ****p < 0.0001.

Subtotal nephrectomy (SNx) animals developed significant proteinuria and high blood pressure, along with renal hypertrophy (Fig. 1a,b,d). Blood urea nitrogen levels were also elevated in SNx animals (Fig. 1c). Moderately affecting body weight (Table 1), treatment of SNx animals with theracurmin was associated with a reduction in kidney weight and an attenuation of systolic hypertension when compared to untreated SNx animals. Systolic blood pressure, although elevated in all SNx animals at 3-week post surgery, was improved over the course of 5 weeks with theracurmin-treatment (Fig. 1a). Differences in urinary protein or blood urea nitrogen levels were not observed between SNx and theracurmin-treated SNx animals.

**Figure 2:**
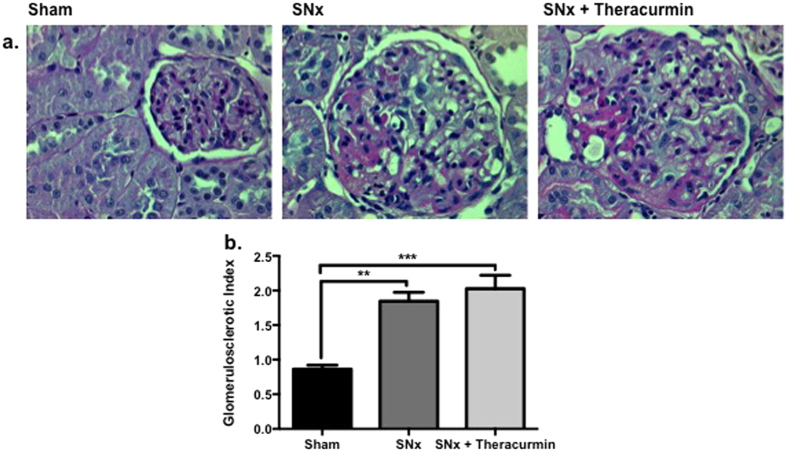
Theracurmin does not impact kidney structure. Representative images of (**a**) PAS-stained kidney sections of sham-operated controls, SNx, and theracurmin-treated SNx animals. (**b**) Quantitative analysis assessing degree of glomerulosclerosis. Data presented as mean ± SEM. **p < 0.01, ***p < 0.001.

Glomerulosclerosis was a prominent feature in SNx animals (Fig. 2a). Sham-operated control animals exhibited normal glomerular morphology, while both SNx groups (untreated and theracurmin-treated animals), exhibited moderate glomerulosclerosis (Fig. 2a). The degree of glomerulosclerosis in the theracurmin-treated animals was similar to that of untreated animals (Fig. 2b).

### Theracurmin improves cardiac structure and function in SNx animals

**Figure 3:**
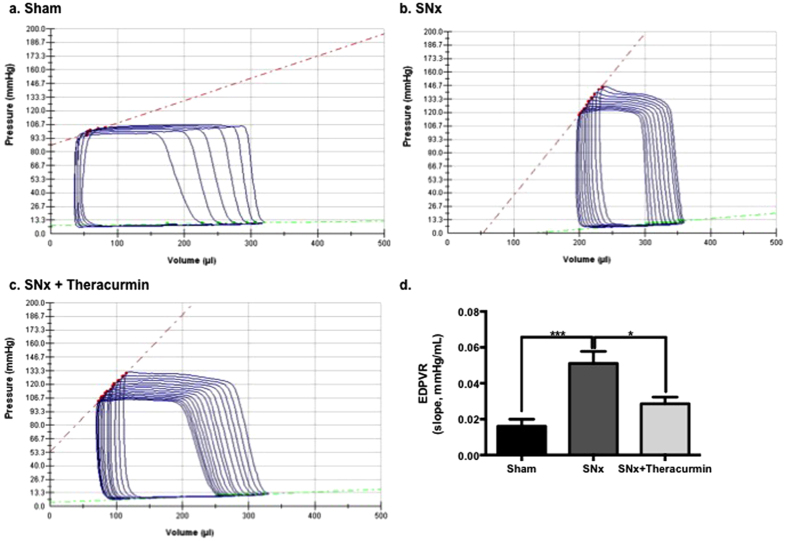
Theracurmin improves diastolic function in SNx animals. Pressure-volume loop analysis of (**a**) sham-operated controls, (**b**) SNx animals, and (**c**) theracurmin-treated animals at eight weeks post surgery. (**d**) Quantitative analysis of PV loops, showing EDPVR from each group. Data presented as mean ± SEM. *p < 0.05, and ***p < 0.001

Subtotal nephrectomy was associated with significant abnormalities in diastolic function, as assessed by pressure-volume loops (Fig. 3). Tau Logistic, a measure of active ventricular relaxation under preload reduction, was significantly prolonged in SNx animals when compared to sham control animals (7.80 ± 0.33 vs. 10.08 ± 1.70; p < 0.001). An increase in the slope of the end diastolic pressure volume relationship (EDPVR), which is an accurate representation of chamber stiffness, was also noted in SNx animals (Fig. 3b,d). Active relaxation (τ Logistics) was not modified in theracurmin-treated animals when compared with untreated SNx animals (10.08 ± 1.70 vs. 10.15 ± 1.65); however, chamber compliance (slope of the EDPVR) was greatly improved (Fig. 3c,d). Differences in measures of systolic function were not observed in sham-operated control animals when compared to SNx animals (Fig. 3a,b).

**Figure 4:**
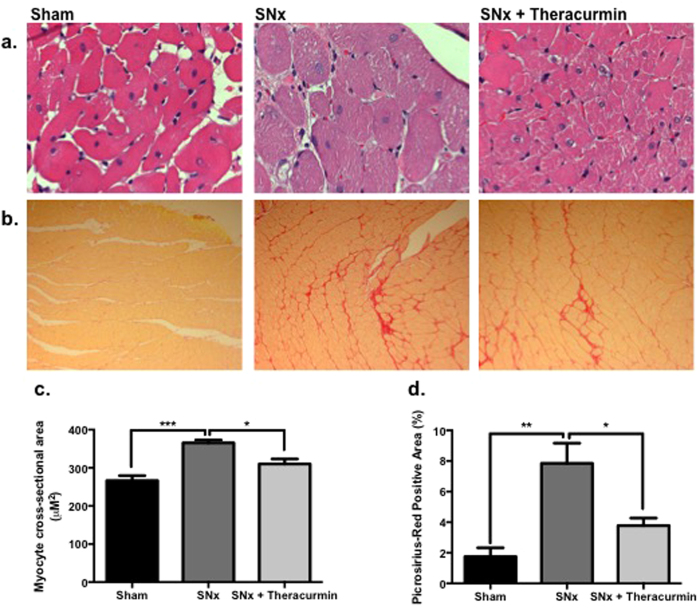
Theracurmin reduces myocyte hypertrophy and cardiac fibrosis. (**a**) H&E and (**b**) Picrosirius-red staining (PSR) of cardiac sections assessing hypertrophy and fibrosis, respectively. Quantitative analysis of (**c**) H&E and (**d**) PSR-stained sections. Data presented as mean ± SEM. *p < 0.05, **p < 0.01, ***p < 0.001

Pathological remodeling—including cardiac fibrosis, myocyte hypertrophy, and wall thickening—was observed in SNx animals (Table 1, Fig. 4). Treatment of SNx animals with theracurmin was associated with a significant reduction in LV weight (Table 1). This observation was corroborated with echocardiographic assessment of cardiac function, which also showed a significant reduction in LV mass in theracurmin-treated animals (Table 1). Left ventricular internal diameter in diastole and systole were lower in SNx animals, with no significant difference between the groups. Anterior and posterior wall thickness in diastole was significantly greater in SNx animals compared to sham-operated animals; with theracurmin-treated animals showing a small, but non-significant reduction in anterior wall thickness. Interstitial fibrosis and myocyte hypertrophy, which are primary mediators of chamber stiffness, were markedly increased in SNx animals when compared to sham-operated animals; but significantly reduced in theracurmin-treated animals (Fig. 4).

### Theracurmin attenuates NLRP3 inflammasome activation

**Figure 5:**
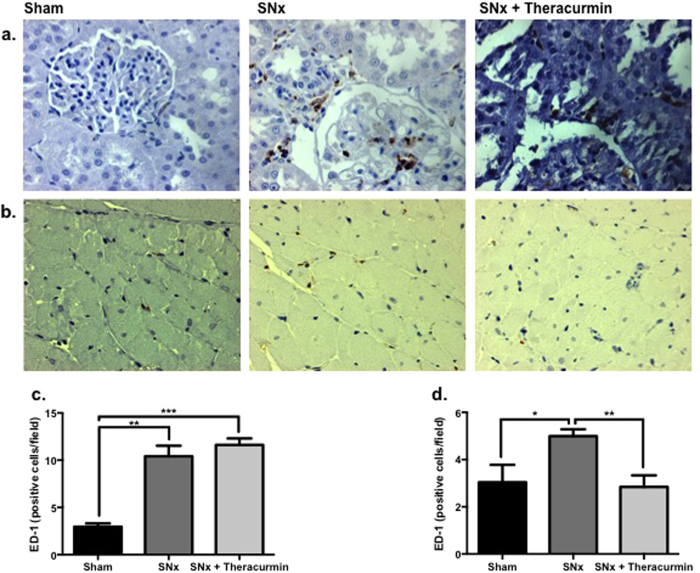
Macrophage infiltration in the kidney and heart. Representative images of (**a**) ED-1 stained kidney sections of sham-operated controls, SNx, and theracurmin-treated animals. (**b**) Macrophage infiltration in the subendocardial zone. (**c**) Quantitation of ED-1 staining in renal and (**d**) cardiac tissue sections. Data presented as mean ± SEM. *p < 0.05, **p < 0.01, ***p < 0.001

The activation of systemic inflammation, which is common in the progression of both renal and cardiac failure, was noted in SNx animals. Macrophages were detected in the tubulointerstitium and to some extent, in the glomerulus of SNx animals (Fig. 5a,c). No significant difference in the number of ED-1 positive cells in the kidney was observed in theracurmin-treated versus untreated SNx animals. In the heart, a significant increase in ED-1 positive cells was observed in the subendocardial zone of SNx animals (Fig. 5b) compared to sham-operated controls. However, in contrast to that seen in the kidney, macrophage infiltration in the hearts of theracurmin-treated animals was lower when compared to untreated SNx animals (Fig. 5b,d).

**Figure 6:**
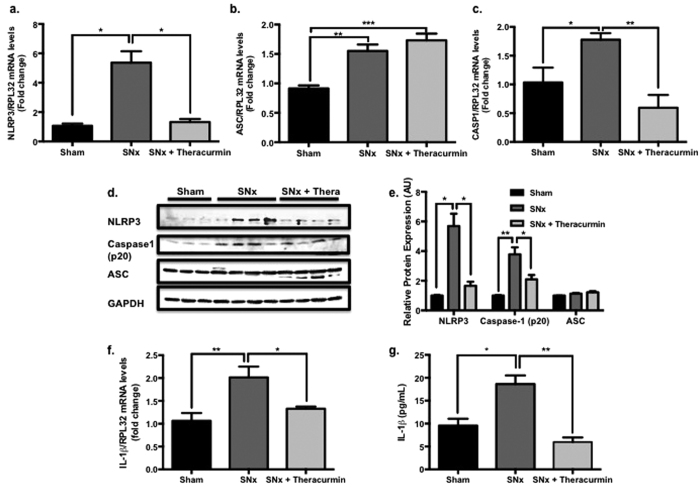
Theracurmin suppresses NLRP3 inflammasome activation. Transcript levels of NLRP3 inflammasome component (**a**) NLRP3, (**b**) ASC, and (**c**) caspase-1 were assessed in all groups. (**d**) Protein expression levels of NLRP3 inflammasome components (lanes 1–3, sham controls; lanes 4–7, SNx; and lanes 8–11, SNx + Theracurmin) and (**e**) quantification data are presented. (**f**) IL-1β expression at the transcript level and (**g**) circulating IL-1β concentration in the serum. Data presented as mean ± SEM. *p < 0.05, **p < 0.01, ***p < 0.001

NLRP3 inflammasome-related cytokines such as IL-1β and IL-18 have been shown to contribute to renal inflammation and fibrosis. As such the expression of key NLRP3 inflammasome components were assessed to determine if the cardio-protective effects of theracurmin noted thus far were mediated through NLRP3 inflammasome modulation. NLRP3 was significantly upregulated at the transcript and protein level in SNx animals when compared to sham-operated control animals (Fig. 6a,d,e). Since the assembly and activation of the NLRP3 inflammasome relies on the adaptor molecule ASC, which interacts with procaspase-1—invariably leading to the autocatalytic activation of caspase-1—to process pro-IL-1β and pro-IL-18 into their active forms, the expression of ASC and caspase-1 were also assessed in all animals. The mRNA level of all components of the NLRP3 inflammasome (NLRP3, ASC, and caspase-1) were upregulated in the hearts of SNx animals compared to sham controls (Fig. 6a,b,c). This was corroborated at the protein level, with the exception of ASC, which remained constant across the three groups (Fig. 6d,e). Treatment of SNx animals with theracurmin significantly reduced the expression of both NLRP3 and caspase-1 in the heart without affecting the expression of the adaptor molecule ASC (Fig. 6a–e). Renal expression of all NLRP3 inflammasome components, while upregulated in SNx animals, was not altered in theracurmin-treated animals (data not shown).

NLRP3 inflammasome activation results in the maturation and secretion of IL-1β. In addition to the reduction of NLRP3 inflammasome components, cardiac IL-1β expression at the transcript level, which was upregulated in SNx animals, was also reduced in theracurmin-treated animals (Fig. 6f). Serum IL-1β concentration was notably elevated in SNx animals (Fig. 6g). However, in theracurmin treatment animals, serum IL-1β levels were comparable to those of sham-operated control animals (Fig. 6g).

### Theracurmin attenuates the expression of pro-fibrotic factors in SNx animals

**Figure 7:**
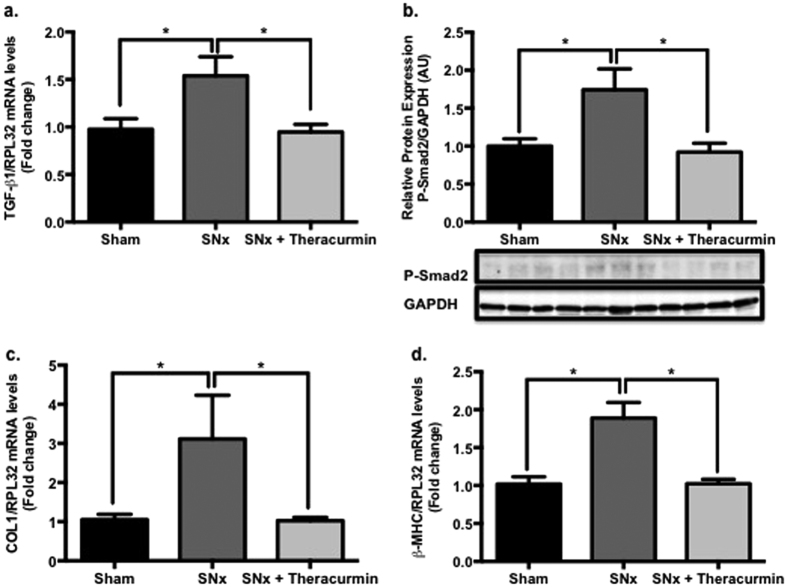
Theracurmin reduces the expression of pro-fibrotic/pro-hypertrophic factors in SNx animals. The expression levels of (**a**) TGF-β1, (**b**) phopho-Smad2, (**c**) collagen type I and (**d**) β-MHC, markers of pathological fibrosis and hypertrophy, were assessed in all animals. Data presented as mean ± SEM. *p < 0.05

Since inflammation is a critical driver of fibrosis, the expression of pro-fibrotic factors was also assessed in all animals. Consistent with the morphometric and histological analysis, the expression of pro-fibrotic and pro-hypertrophic factors at the transcript level was elevated in the heart of SNx animals, when compared to sham-operated controls (Fig. 7a,c,d). Treatment of SNx animals with theracurmin reduced the expression of TGF-β1, collagen type I, and β-MHC in the heart when compared to untreated SNx animals. Smad2 phosphorylation, which is indicative of canonical TGF-β signaling activation, was observed to be higher in SNx animals when compared to sham controls, but lower in theracurmin-treated aniamls (Fig. 7b). Renal expression of these pro-hypertrophic/pro-fibrotic markers was elevated in SNx animals but was not altered in theracurmin-treated animals (data not shown).

## Discussion

In the present study, we demonstrate that theracurmin—a novel formulation of curcumin—possesses anti-inflammatory and anti-fibrotic activity in the hearts of rats after the induction of renal injury via 5/6 subtotal nephrectomy. The cardio-protective effects of theracurmin are achieved, in part, through blood pressure modulation and the suppression of NLRP3 inflammasome activation in the heart.

In alignment with previous studies, the remnant kidney model (5/6 SNx) used in this study developed key characteristic of renal injury typically observed in humans, namely the development of hypertension, proteinuria, glomerulosclerosis and macrophage infiltration. We demonstrate that treatment with theracurmin—a novel formulation of curcumin with a 30-fold higher bioavailability than conventional curcumin—modestly reduced systolic blood pressure levels, but did not attenuate the progression of proteinuria nor impact damage to renal structure or function in the remnant kidney. Although studies employing the use of conventional curcumin have reported an improvement in systolic blood pressure levels in the remnant kidney model^13^,^14^,^15^, these studies also reported a significant reduction in proteinuria, glomerulosclerosis and tubulointerstital injury. Aside from differences in the composition of the therapy, a major difference between these studies and the present study is the timeline of treatment. In our study, the animals received theracurmin for 5 consecutive weeks, following the development of 3 weeks of progressive renal injury, while the aforementioned studies relied on a 7-week regimen. It is plausible that had we extended our treatment timeline, theracurmin may have had a noticeable impact on renal function in addition to the observed reduction in blood pressure levels.

Blood pressure control is generally regarded as an effective strategy to circumvent the progression of chronic kidney disease and the ensuing cardiovascular complications^16^,^17^,^18^. Studies in humans and experimental models of chronic kidney disease have shown that blood pressure reduction leads to an improvement in proteinurea and in the degree of glomerulosclerosis and interstitial fibrosis^16^,^17^,^19^,^20^,^21^. In this study, although theracurmin reduced blood pressure levels of SNx animals, this effect did not translate to an improvement in renal structure or function. This is likely because theracurmin treated animal remained hypertensive despite the significant, albeit modest, blood pressure reduction observed in relation to their untreated counterparts. Additionally, given the severity of the SNx model, which is characterized by significant renal damage, it is reasonable to infer that possible changes to renal structure mediated by theracurmin at the chosen time point may have been too subtle to detect or to be accompanied by functional changes.

The presence of chronic kidney disease confers a markedly increased risk of cardiovascular death. In fact, patients with chronic kidney disease often die from cardiovascular complications before progressing to end-stage kidney failure^1^,^22^; with studies reporting myocardial fibrosis and left ventricular hypertrophy as consistent findings in biopsies of patients with chronic kidney disease^3^,^23^,^24^. In accordance, the remnant kidney model used in this study consistently demonstrated signs of cardiac fibrosis, hypertrophy, and diastolic dysfunction. Our pressure-volume loop analysis data and histological findings showed a significant improvement in diastolic function with theracurmin treatment, along with a pronounced reduction in myocyte size and interstitial fibrosis—a dominant feature of chronic kidney disease-associated structural myocardial remodeling. This is in accordance with the findings of a double-blinded clinical trial study of 68 hypertensive patients with left ventricular hypertrophy, which showed that theracurmin improved diastolic function in hypertensive patients via a 12% reduction in left ventricular stiffness^25^.

It is well established that inflammation plays a critical role in the progression of chronic kidney disease^26^. Along the same lines, growing evidence suggests that conventional curcumin possesses strong anti-oxidant and anti-inflammatory properties that make it an effective compound for use in both chronic and acute models of inflammation. Indeed, the use of curcumin has been shown to attenuate NF-κB activation and reduce macrophage infiltration in rats after unilateral ureteral obstruction^27^. Curcumin usage has also been shown to ameliorate renal failure in subtotal nephrectomy rats by antagonizing TNFα^13^. In light of these studies, it is important to explore the potential anti-inflammatory effects of theracurmin to deduce its potential application in the clinic. In this study, we show for the first time that a novel formulation of curcumin with improved bioavailability improves cardiac function by suppressing NLRP3 inflammasome activation and mature IL-1β release in rat model of chronic kidney disease.

The NLRP3 inflammasome is an innate proteolytic complex that is activated by a variety of danger associated molecular patterns or cellular contents that are inappropriately released in the event of an injury^28^. In the event of an injury, NLRP3 proteins oligomerize and recruit the adaptor protein ASC and the protease caspase-1 to form a complex termed “the inflammasome”^29^. The formation of the inflammasome induces caspase-1 activation, which leads to the processing of pro-IL-1β and pro-IL-18 from their inactive forms into mature secreted cytokines^28^. Recently, inflammasome-derived cytokines IL-1β and IL-18 have been shown to modulate the cardiac remodeling process after injury and depress myocardial function^30^,^31^,^32^,^33^. Accordingly, it is not surprising that in our remnant kidney model, an upregulation of cardiac NLRP3 inflammasome components were observed in addition to a significant increase of circulating IL-1β levels. Indeed, Vilaysane *et al*. recently reported an increase in NLRP3 mRNA expression levels using both human renal biopsies and an experimental model of chronic kidney disease^9^. Although they did not explore cardiac expression of NLRP3 inflammasome components in their study, their findings along with ours strongly suggest a role for NLRP3 in renal injury and cardiovascular dysfunction. The positive correlation between cardiac fibrosis and NLRP3 expression suggests that the cardio-protective benefits of theracurmin may, in part, be due to its effect on lowering cardiac NLRP3 expression and circulating IL-1β levels.

In summary, the results of the present study are consistent with the notion that inflammation plays an important role in the progression of chronic kidney disease and cardiovascular complications. Treatment with theracurmin, used for the first time in the remnant kidney model, attenuated cardiac fibrosis and NLRP3 inflammasome activation without impacting kidney structure or function. Overall, theracurmin, and other modulators of cardiac NLRP3 inflammasome, may present as novel therapeutic options for the treatment of cardiovascular events in the setting of chronic kidney disease.

## Methods

### Animal Model

Animal studies were conducted with the approval of the St. Michael’s Hospital Animal Ethics Committee, in accordance with the Guide for the Care and Use of Laboratory Animals (NIH publication no. 85–23, revised 1996). All animals received normal rat chow and drinking water *ad libitum*. Animals were also housed in a stable environment maintained at a temperature of 22 ± 1 °C, with a 12-hour light/dark cycle.

### Subtotal Nephrectomy

Fifty-six male Sprague-Dawley rats were randomized to undergo subtotal nephrectomy (SNx) or sham surgery. The sham control group (n = 8) underwent sham surgery consisting of the removal of the right kidney, followed by the manipulation of the left kidney before wound closure. The remaining 48 animals underwent SNx, which was performed by the removal of the right kidney and infarction of approximately 2/3 of the remaining left kidney by selective ligation of the posterior branch and the anterior segmental artery of the anterior branch^34^. At three weeks post surgery, the surviving SNx animals (n = 42) were randomized into two groups, with one group (n = 18) receiving 100 mg/kg/day of theracurmin (Theravalues Corporation, Chiyoda-ku, Tokyo, Japan) and the other group (n = 24) receiving vehicle (saline) by oral daily gavage for 5 consecutive weeks. At end study, there were 8 shams, 16 SNx, and 15 SNx + theracurmin animals remaining.

### Renal function

Animals were weighed weekly. Systolic blood pressure levels were measured in conscious animals using a tail-cuff plethysmogragh attached to a pulse transducer. Animals were housed individually in metabolic cages for 24 hours at baseline, 3 weeks post surgery, and at end-study. After 24 hours in metabolic cages, 3 mL aliquot of urine was collected from the 24-hour urine sample and stored in −80 °C for subsequent analysis. Urine albumin and creatinine levels were measured by the Department of Pathology, Toronto General Hospital, Toronto ON, Canada.

### Cardiac function

Echocardiography was performed in anesthetized animals with a high frequency imaging system (Vevo 2100, Visualsonics Inc.). All parameters were assessed using an average of 3 consecutive cardiac cycles.

Cardiac catheterization was performed as previously published^35^. In brief, a 2F miniaturized combined conductance catheter-micromanometer (Model SPR-838, Millar instruments, Houston, Tex) was inserted into the carotid artery to obtain aortic blood pressure, then advanced into the left ventricle until stable PV loops were obtained^36^. Data were then acquired under steady state conditions and during preload reduction. The following functional parameters were then calculated (Millar analysis software PVAN 3.4): end-diastolic volume, end-diastolic pressure, end-systolic pressure, and the slope of the end-diastolic pressure volume relationship.

### Histology

Paraffin-embedded renal and cardiac tissue sections, about 4 mm thick, were examined in all animals. Picrosirius red stained heart sections were used to quantify matrix accumulation; analysis was performed using computer-assisted image analysis in a blinded fashion, as previous reported^37^. Myocyte hypertrophy was assessed using haematoxylin and eosin stained sections; method was adapted from Kai and colleagues^38^ and performed as previously reported^39^.

Immunohistochemical analysis of macrophage infiltration was performed using a mouse anti-rat CD68/ED-1 antibody (1:100, MCA341A488, Bio-Rad, Mississauga ON) in a 1:100 dilution. ED-1 positive cells were counted in approximately 8–10 randomly selected high fields per section at a magnification of x400 and results were presented as the average number of ED-1 positive cells per field.

Periodic acid Schiff (PAS)-stained sections were examined using a Carl Zeiss microscope attached to an AxioCamMRc5 digital camera (Carl Zeiss, North Ryde, NSW, Australia). Approximately 50 glomeruli were randomly selected and the degree of glomerular damage assessed using a semi-quantitative scoring method/graded scale of 0 to 4, as previously described^40^. Grade 0, normal glomeruli; grade 1, sclerotic area up to 25% (minimal sclerosis); grade 2, sclerotic area up to 25–50% (moderate sclerosis); grade 3, sclerotic area up to 50–75% (moderate to severe sclerosis); grade 4, sclerotic area up to 75–100% (severe sclerosis). An observer masked to the treatment groups performed the glomerulosclerotic index analysis.

### Serum IL-1β concentration

Serum concentration of the pro-inflammatory cytokine IL-1β was determine in all animals using the IL-1F2 Quantikine enzyme-linked immunosorbent assay kit (Cat. RLP00; R&D, Minneapolis, MN) in accordance to the manufacturer’s instructions. A microplate reader was used to measure absorbance at 450 nm.

### Quantitative Real Time PCR

Total RNA from the hearts of all animals was extracted with Trizol^®^ (Invitrogen, Carlsbad CA) and precipitated with isopropyl alcohol. Complementary DNA (cDNA) was synthesized with the High-Capacity cDNA Reverse Transcription kit (Applied Biosystems, Foster City, CA) and subjected to quantitative RT-PCR with the ABI Prisim 7000 Sequence Detection System (Applied Biosystems, Foster City, CA) according to the manufacturer’s instructions, as previously described^39^. Briefly, SYBR^®^ green master mix (Applied Biosystems, Foster City, CA) was mixed with both forward and reverse primers for RPL32, NLRP3, ASC, caspase-1, IL-1β, TGF-β1, collagen type 1, and β-MHC according to the manufacturers instructions. Primer sequences were as followed: RPL32 (forward) TGAAGCCCAAGATCGTCAAAAAG, RPL32 (reverse) GCACAGTAAGATTTGTTGCACATC; NLRP3 (forward) GCTGCTCAGCTCTGACCTCT, NLRP3 (reverse) AGGTGAGGCTGCAGTTGTCT; ASC (forward) GCAATGTGCTGACTGAAGGA, ASC (reverse) TGTTCCAGGTCTGTCACCAA; Caspase-1 (forward) GGAGGGAATATGTGGGATCA, Caspase-1 (reverse) CCCTCTTCGGAGTTCCCTAC; IL-1β (forward) CTGGATGCTCTCATCTGGAC, IL-1β (reverse) AACTGTCCCTGAACTCAACTG; TGF-β1 (forward) CACCCGCGTGCTAATGGT, TGF-β1 (reverse) TGTGTGATGTCTTTGGTTTTGTCA; Collagen type I (forward) TGCCGATGTCGCTATCCA, Collagen type I (reverse) TCTTGCAGTGATAGGTGATGTTCTG; β-MHC (forward) GTGCCAAGGGCCTGAATGAG, β-MHC (reverse) GCAAAGGCTCCAGGTCTGA.

### Western blotting

Total protein was extracted from all animals using ice-cold RIPA buffer (Thermo Scientific, Rochester NY) containing a Halt^®^ protease inhibitor cocktail (Thermo Scientific, Rochester NY) and quantified using the Bio-Rad Protein Assay Reagent. Protein lysates (30 μg) were separated by SDS-PAGE gel and transferred onto nitrocellulose membranes, which were blocked with 5% skim milk at room temperature for 2 hours, and incubated overnight at 4 °C with the following antibodies: GAPDH (Santa Cruz, sc-25778), NLRP3 (R&D, 7578), ASC (Santa Cruz, sc-22514), Caspase-1 (Millipore, AB1871), and phospho-Smad2 (Cell Signaling, 3108). Following the overnight incubation, anti-rabbit IgG (Cell Signaling, 7074P2) and anti-mouse IgG (Cell Signaling, 7076P2) conjugated to horseradish peroxidase were used as secondary antibodies and signal was visualized with an enhanced chemiluminescence detection system (Bio-Rad, Rochester NY) and quantified by densitometry.

### Statistics

Data are expressed as mean ± SEM. Statistical significance was determined by a non-parametric Kruskal-Wallis test with Dunn’s correction. Analyses were performed using GraphPad Prism 7 (GraphPad Software Inc., San Diego, CA). A p-value < 0.05 was considered as statistically significant.

## Additional Information

**How to cite this article**: Bugyei-Twum, A. *et al*. Suppression of NLRP3 Inflammasome Activation Ameliorates Chronic Kidney Disease-Induced Cardiac Fibrosis and Diastolic Dysfunction. *Sci. Rep.* **6**, 39551; doi: 10.1038/srep39551 (2016).

**Publisher's note:** Springer Nature remains neutral with regard to jurisdictional claims in published maps and institutional affiliations.

## Figures and Tables

**Table 1:** 

Morphometric Characteristics of Animals			
	Sham	SNx	SNx + Theracurmin
N	8	15	11
Body weight (g)	665 ± 35	588 ± 10^†^	489 ± 19^‡^
Tibia length (mm)	41 ± 0.5	43 ± 0.4	40 ± 0.4^‡^
Lung weight (g)	1.88 ± 0.06	1.91 ± 0.06	1.77 ± 0.07
Heart weight (g)	1.58 ± 0.07	2.03 ± 0.08^††^	1.90 ± 0.05
LV weight (g)	1.18 ± 0.04	1.51 ± 0.05^†^	1.38 ± 0.04
LV weight/tibia length (g/mm)	2.87 ± 0.07	3.76 ± 0.11^†^	3.39 ± 0.10^‡^
**Echocardiographic Parameters**			
	**Sham**	**SNx**	**SNx + Theracurmin**
N	8	13	11
LV Mass Corrected (mg)	1523 ± 130	2098 ± 168^†^	1515 ± 100^‡^
LVIDd (mm)	9.00 ± 0.28	8.66 ± 0.20	8.26 ± 0.23
LVIDs (mm)	4.27 ± 0.43	3.94 ± 0.30	3.46 ± 0.30
LVAWd (mm)	2.30 ± 0.09	2.85 ± 0.15^†^	2.66 ± 0.07
LVPWd (mm)	2.33 ± 0.09	3.02 ± 0.13^†^	3.01 ± 0.14
Ejection Fraction (%)	53 ± 3.7	54 ± 2.5	58 ± 3.2
Fractional Shortening (%)	81 ± 3.2	83 ± 2.3	85 ± 2.4

Values are expressed as mean ± SEM. LV, left ventricular; SNx, subtotal nephrectomy. ^†^p < 0.05, ^††^p < 0.01 compared with sham; ^‡^p < 0.01, compared with SNx.

Values are expressed as mean ± SEM. SNx, subtotal nephrectomy. LVIDd, left ventricular internal diameter in diastole; LVIDs, left ventricular internal diameter in systole; LVAWd, left ventricular anterior wall thickness in diastole; LVPWd, left ventricular posterior wall thickness in diastole; ^†^p < 0.05 compared with sham, ^‡^p < 0.05, compared with SNx.
